# Use of an Artificial Neural Network for Tensile Strength Prediction of Nano Titanium Dioxide Coated Cotton

**DOI:** 10.3390/polym14050937

**Published:** 2022-02-26

**Authors:** Nesrine Amor, Muhammad Tayyab Noman, Adla Ismail, Michal Petru, Neethu Sebastian

**Affiliations:** 1 Department of Machinery Construction, Institute for Nanomaterials, Advanced Technologies and Innovation (CXI), Technical University of Liberec, Studentská 1402/2, 461 17 Liberec, Czech Republic; michal.petru@tul.cz; 2 Laboratory of Signal Image and Energy Mastery (SIME, LR 13ES03), Electrical Engineering Department, University of Tunis, ENSIT, Tunis 1008, Tunisia; adla.ismail1@gmail.com; 3 Institute of Organic and Polymeric Materials, National Taipei University of Technology, No. 1, Section 3, Zhongxiao East Road, Taipei 106, Taiwan; sneethu@mail.ntut.edu.tw

**Keywords:** artificial neural network, tensile strength, titanium dioxide nanoparticles

## Abstract

In this study, an artificial neural network (ANN) is used for the prediction of tensile strength of nano titanium dioxide (TiO_2_) coated cotton. The coating process was performed by ultraviolet (UV) radiations. Later on, a backpropagation ANN algorithm trained with Bayesian regularization was applied to predict the tensile strength. For a comparative study, ANN results were compared with traditional methods including multiple linear regression (MLR) and polynomial regression analysis (PRA). The input conditions for the experiment were dosage of TiO_2_, UV irradiation time and temperature of the system. Simulation results elucidated that ANN model provides high performance accuracy than MLR and PRA. In addition, statistical analysis was also performed to check the significance of this study. The results show a strong correlation between predicted and measured tensile strength of nano TiO${}_{2}$-coated cotton with small error values.

## 1. Introduction

The widespread applications and versatile properties of composite materials make them powerful in materials science. TiO${}_{2}$ in nano forms (nanorods, nanoparticles, nanosheets, nanowires, nanoflowers) have shown its potential in various industries including textiles as a coating material. The properties that make TiO${}_{2}$ unique are chemical stability, photocatalytic activity and non-toxicity [1]. In recent years, researchers have coated nano TiO${}_{2}$ on textile substrates to make functional textiles [2,3]. In an experimental study, Noman et al. synthesized and coated TiO${}_{2}$ nanoparticles on cotton fabric by UV light and investigated the tensile behaviour and stabilization of nanoparticles in real conditions [4]. However, as well as we know, there is no such study available in which theoretical evaluation of tensile strength and stabilization of nanoparticles coated cotton were performed, and a comparison of ANN, MLR and PRA was drawn for better efficiency. Therefore, in this work, a prediction model (based on a comparative study of ANN, MLR and PRA) is designed via machine learning methods for theoretical evaluation of tensile strength as well as the stabilization of nano TiO${}_{2}$ on cotton fabric. The designed model works in the following manner i.e., correlates the actual response with the process variables, evaluates the predicted response and indicates the better approach. ANN models are the widely used machine learning tools for prediction and classification of real-world applications e.g., textile processes [5,6], computer vision [7], materials engineering [8,9] and biomedical engineering [10,11,12]. ANN has great potential for prediction from input variables, especially when an unknown mathematical relationship exists between input and output variables [13,14,15].

Tensile strength is an important indicator for the mechanical performance of fibrous materials. Lu et al. applied ANN and MLR models based on acoustic emission detection to predict the breaking strength of wool fiber [16]. The results showed that there is a strong correlation between actual and predicted values of wool strength in terms of coefficient of determination under both ANN and MLR models. However, ANN model provided higher accuracy and less error than MLR. Gayatri et al. employed ANN to predict the tensile strength of hybrid composites that made of carbon fiber, epoxy resin and glass fiber [17]. Experimental results showed that ANN was able to predict the tensile strength parameters with high accuracy as compared to MLR. Mishra predicted the yarn strength utilization during the fabrication of cotton fabric using ANN model [18]. The experimental work showed that there was an increase in the percentage of yarn strength utilization with an increase in yarn number in both directions, however, a decrease in float length and crimp percentage was also observed. In another study, Malik et al. used the back propagation ANN model to predict the tensile properties of uneven and even yarns extracted from polyester-cotton blend [19]. The results showed that ANN was able to predict the tensile properties with lower error values. Altarazi et al. used ANN, stochastic gradient descent (SGD), k-nearest neighbors (kNN), logistic regression (LoR), random forest (RF), regression analysis, decision tree (DT), support vector machine (SVM) and AdaBoost (AB) algorithms to classify and predict tensile strength of polymeric films of different compositions [20]. Testing results showed that the best prediction accuracy was obtained with SVM algorithm and all used algorithms provided an excellent classification for sorting films into non-conforming ad conforming parts. Erbil et al. applied ANN and MLR algorithms to predict tensile strength of ternary blended open-end rotor yarns [21]. They used stepwise MLR and ANN models, trained with Levenberg–Marquardt backpropagation function. The results showed that ANN model outperformed MLR in the prediction accuracy of elongation at break and breaking strength. Breuer et al. used ANN to predict the short fiber composite properties using RVE database [22]. The prediction of the elastic properties of short fiber reinforced plastics by ANN has been compared with additional finite element results. ANN was able to predicted the stiffness of short fiber reinforced plastics. Wang et al. implemented ANN to predicted the tensile strength of ultrafine glass fiber felts [23]. The tensile strength was modelled based on the mean diameter of fibers, resin content and bulk density. The results demonstrated that ANN model provides excellent prediction accuracy with fewer errors. In another experimental study, Liu et al. used ANN model to predict the tensile behavior of hybrid fiber reinforced concrete (HFRC) consists of slag power and fly ash [24]. Simulation results revealed that ANN model provides better prediction accuracy compared to other classic method (Equation-based model) in terms of tensile strength, tensile stress-strain curve and strain corresponding to tensile strength.

Recently, ANN has shown its effectiveness in the prediction of not only tensile strength but many other parameters including dye removal efficiency and functional properties of composites [25,26,27,28]. ANN has the advantages of high nonlinearity resolution, self-learning and mapping capability between input and output variables without introducing a mathematical model between nonlinear data. Therefore, investigating the accuracy of ANN model for tensile strength prediction and draw a performance comparison of ANN with MLR and PRA using statistical analysis provides significant values to this study.

## 2. Material and Methods

### 2.1. Material and Experimental Design

Plain weave cotton fabric with 115 g·m^−2^ fabric mass was used as received from industry. Total 15 samples were prepared and the experimental design under different dosage of TiO${}_{2}$, temperature and UV irradiation time is presented in Table 1.

### 2.2. Artificial Neural Network

**Figure 1:**
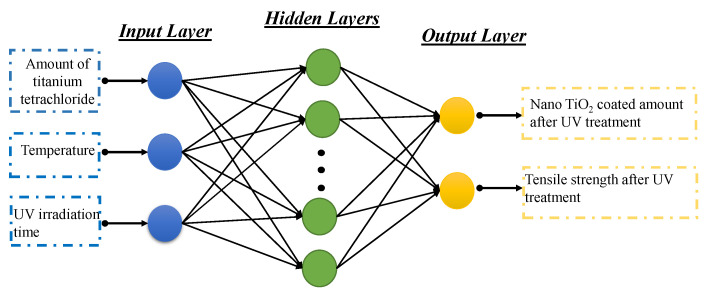
ANN model for the prediction of nano TiO${}_{2}$ coated cotton and tensile strength of coated cotton after UV treatment.

ANN models have been extensively used for the prediction of functional behavior of fibrous materials. The configuration of a back-propagation ANN has been adopted in this work as presented in Figure 1. This configuration is composed of input layers, hidden layers and output layers. In the present case, the amount of titanium tetrachloride, temperature and UV irradiation time were selected as input variables, whereas, TiO${}_{2}$ NPs coated amount and tensile strength were chosen as output variables.

Generally, ANN method are used to develop models for non-linear problems to predict output dependent variables $y=[y_{1},\cdots ,y_{t}]$ using independent input variables $x=[x_{1},\cdots ,x_{l}]$ from their training values [29,30]. The obtained results significantly depend on weights $w=[w_{1},\cdots ,w_{l}]$. The input variables follow a forward path where each input is multiplied by its corresponding synaptic weight and summed up. The relationship between input layer and output layer of ANN model can be expressed by the following equation [30]:

(1) $$y=\varphi \left(\sum_{j}w_{j}*x_{j}+b\right)$$

where, *y* indicates the target (output). φ is the activation function and the most common is sigmoid activation function. $x_{i}$ indicates the selected *i*th input. $w_{i}$ indicates the *i*th weight. *b* represents a constant bias added to the weighted sum. Training of any ANN model is the most important step. The purpose of this training is to optimized the output by minimizing the error between actual and predicted output. After every iteration *k*, the predicted outputs are compared with the actual outputs by computing the error according to Mean Absolute Percentage Error (*MAPE*) method as shown below [31]:

(2) $$MAPE=\frac{1}{N}\Sigma_{i=1}^{N}\Sigma_{j=1}^{n}\left|\frac{y_{ij}-{\hat{y}}_{ij}}{y_{ij}}\right|\;\;\;where\;\;i=1,\cdots ,N\;j=1,\cdots ,n.$$

**Figure 2:**
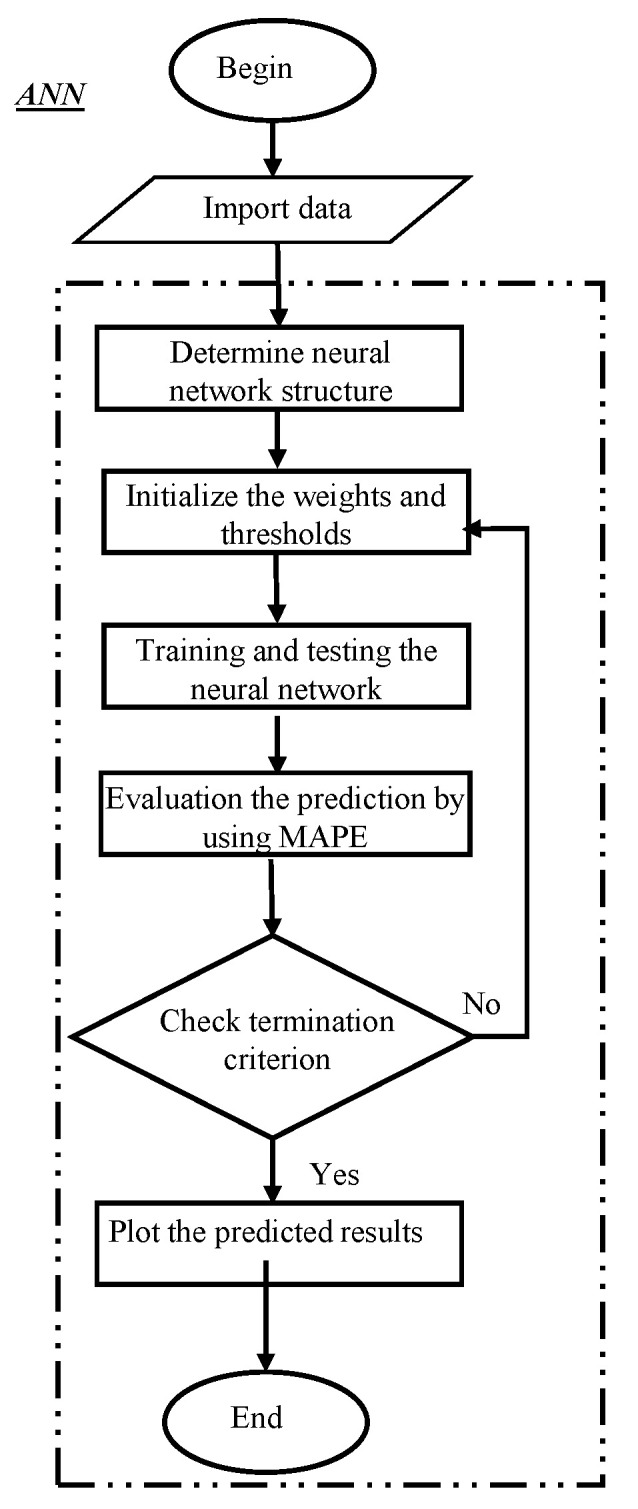
The flowchart of ANN model.

Here, *n* is the number of output nodes and *N* represents the number of training samples. Figure 2 shows the flowchart of ANN model that describes the main steps for prediction process. ANN models and their training process are thoroughly explained in literature [32,33,34].

## 3. Results and Discussion

### 3.1. Structural Analysis

**Figure 3:**
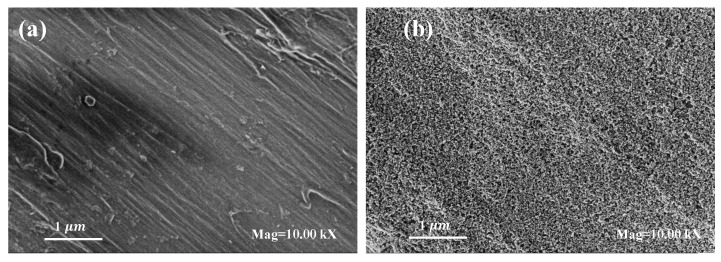
SEM images of cotton fabric: (**a**) untreated sample; (**b**) Nano TiO${}_{2}$-coated sample.

The morphology and topography of uncoated and nano TiO${}_{2}$-coated cotton fabrics were investigated by UHR-SEM (ultrahigh-resolution scanning electron microscopy by Zeiss Ultra Plus, Carl Zeiss Meditec AG, Jena, Germany) analysis as illustrated in Figure 3. Figure 3a shows a smooth and clean surface of untreated cotton whereas Figure 3b shows a huge cluster of nano TiO${}_{2}$ incorporated on cotton as a homogeneous layer.

### 3.2. Analysis of the Proposed ANN Model

**Figure 4:**
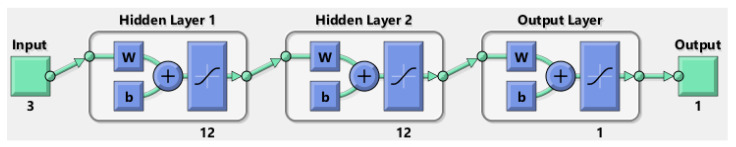
Experimental architecture of the proposed backpropagation ANN model.

We used ANN model to predict nano TiO${}_{2}$ coated amount and tensile strength of cotton after UV treatment. After several trials, we found that the best prediction results for both outputs were obtained by ANN model one input layer, two hidden layers and one output layer, where the number of both hidden layers nodes is 12. In the ANN model, the best choice of a transfer function ensures the best accuracy of predicted results. Therefore, we adopted the use of tansig function type in this work. The training process in the proposed ANN model was based on the use of Bayesian regularization backpropagation algorithm (trainbr). The proposed back-propagation ANN model is presented in Figure 4. For used database, 85% of the data was devoted for training process whereas remaining 15% was designed for testing process. The parameters of training the network are illustrated in Table 2.

**Figure 5:**
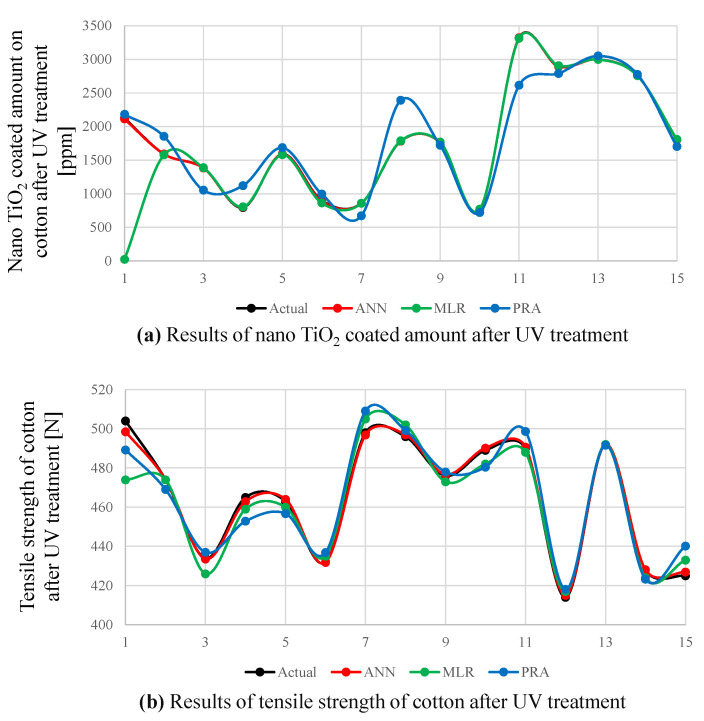
(**a**) The actual and the predicted values of nano TiO${}_{2}$ coated amount after UV treatment under ANN, MLR and PRA. (**b**) The actual and the predicted values of tensile strength of cotton after UV treatment under ANN, MLR and PRA.

**Figure 6:**
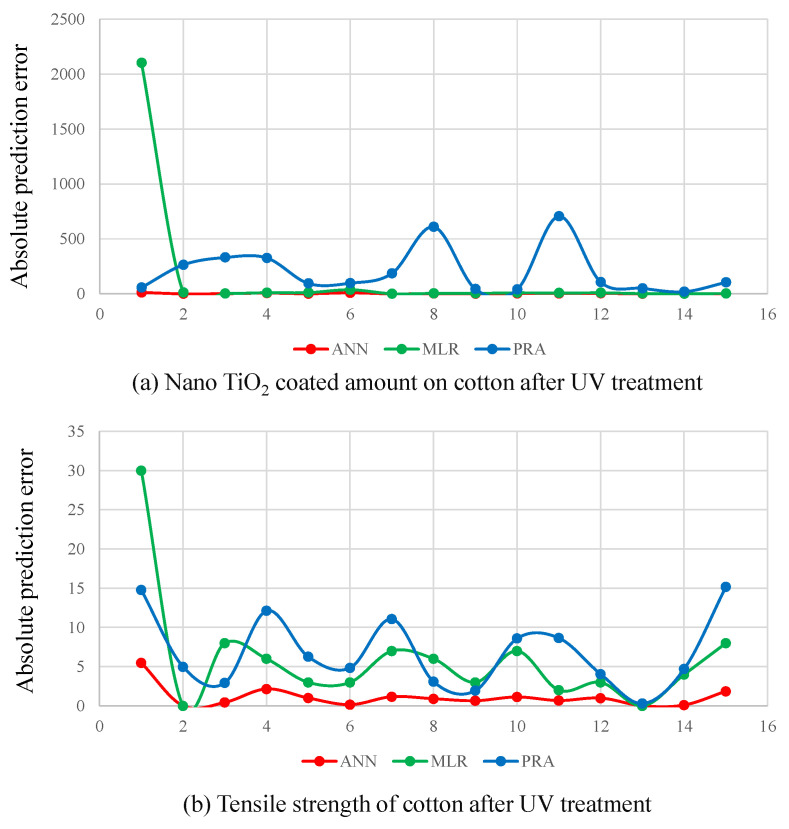
(**a**) Absolute prediction error ($\left|y-\hat{y}\right|$) of nano TiO${}_{2}$ coated amount after UV treatment under ANN, MLR and PRA. (**b**) Absolute prediction error of tensile strength of cotton after UV treatment under ANN, MLR and PRA.

To confirm the accuracy of proposed ANN model, the obtained results were compared with MLR and PRA. The prediction results of all outputs under ANN, MLR and PRA models are illustrated in Figure 5. The absolute prediction errors ($\left|y-\hat{y}\right|$) for both outputs under ANN, MLR and PRA are presented in Figure 6. Figure 6a clearly shows that PRA has the higher errors values for the prediction of nano TiO${}_{2}$ compared to ANN and MLR, especially in values numbers 8 and 11. We also observed that ANN provides slightly better results than MLR, except for the first predicted value where MLR has an error burst. Figure 6b illustrates that ANN outperforms both MLR and PRA for the prediction of tensile strength.

We evaluated the accuracy and performance of ANN, MLR and PRA models through several methods including mean squared error (MSE), mean absolute error (MAE), mean absolute percentage error (MAPE), root mean squared error (RMSE) and coefficient of determination $R^{2}$. All these methods are thoroughly explained in the previous literature [14,15]. The computed prediction errors values by MAE, MSE, RMSE, MAPE and coefficient of determination $R^{2}$ for both outputs under ANN, MLR and PRA models are presented in Table 3. It is revealed from these results that ANN provides excellent prediction accuracy and lower prediction error as compared to MLR and PRA for both outputs.

**Figure 7:**
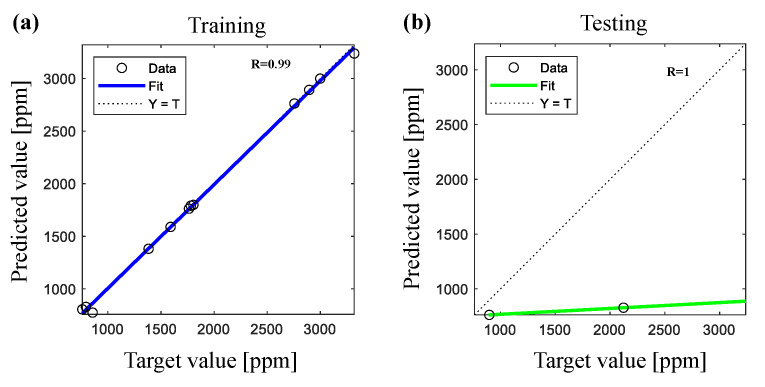
Correlation coefficient between actual and predicted values of nano TiO${}_{2}$ coated amount after UV treatment using ANN during (**a**) training and (**b**) testing processes.

**Figure 8:**
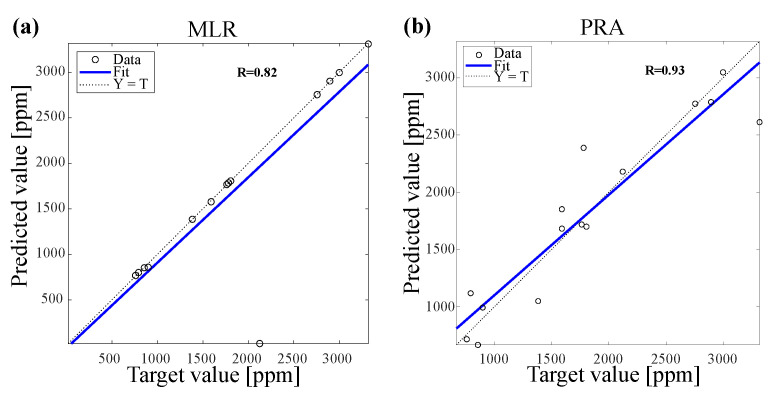
Correlation coefficient between actual and predicted values of nano TiO${}_{2}$ coated amount after UV treatment by using (**a**) MLR and (**b**) PRA.

Figure 7 shows the correlation coefficient R-value between the predicted and the measured values of nano TiO${}_{2}$ coated amount using ANN during training and testing processes of all data sets. Figure 8 illustrates the correlation coefficient for MLR and PRA models. We noticed that the correlation coefficient obtained by ANN (R=99% during training and R=100% during testing) was higher than the correlation coefficient obtained by MLR (R=82%) and PRA (R=93%).

**Figure 9:**
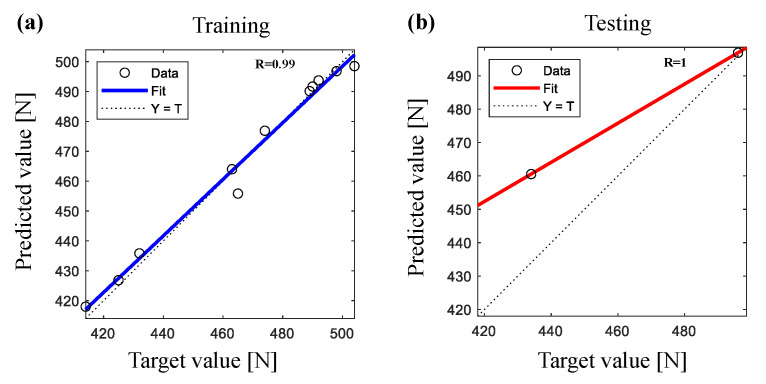
Correlation coefficient between actual and predicted values of tensile strength of cotton after UV treatment using ANN during (**a**) training and (**b**) testing processes.

**Figure 10:**
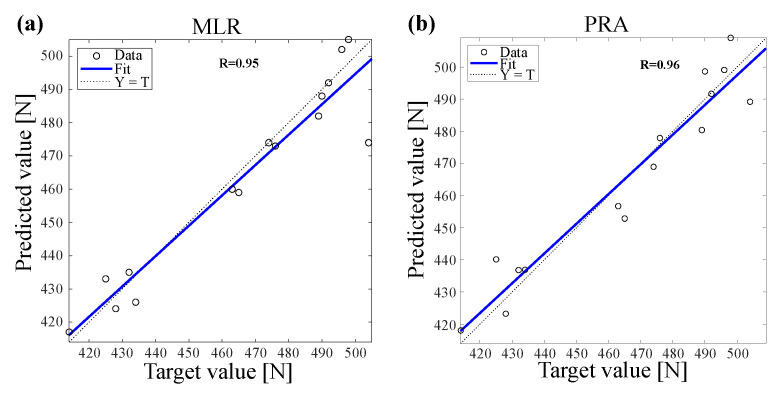
Correlation coefficient between actual and predicted values of tensile strength of cotton after UV treatment by using (**a**) MLR and (**b**) PRA.

The correlation coefficient between the predicted and the actual values of tensile strength using ANN model are presented in Figure 9 and MLR and PRA models are presented in Figure 10. The results revealed that ANN shows higher correlation coefficients than MLR and PRA (ANN R=99% during training, R=100% during testing, MLR R=95% and PRA R=96%) that provides excellent correlation between actual and predicted values for all used models. However, ANN model showed higher correlation coefficient values for both outputs that ensures the effectiveness and high prediction accuracy of ANN model.

A statistical analysis (ANOVA test) was conducted to test the statistical significance of input and output variables [35,36,37,38]. The results for both outputs were tested by One-way ANOVA to check its reliability using ANN, MLR, PRA and experiment values. Table 4 illustrates the results of ANOVA test for both outputs obtained by ANN, MLR, PRA and experimental work. We noticed that the proposed ANN model provides lowest *p*-value for both outputs that means ANN is more statistically significant as compared to experimental values, MLR and PRA.

## 4. Conclusions

In this paper, tensile behaviour of TiO${}_{2}$-coated cotton was predicted with ANN, MLR and PRA models. The proposed ANN model showed much better results than MLR and PRA models. Simulation results showed that ANN has lower error than MLR and PRA in term of MAE, MSE, RMSE and MAPE, and has higher prediction accuracy than MLR and PRA as indicated by coefficient of determination. Therefore, it is revealed that ANN is more efficient prediction tool as compared to MLR and PRA. In addition, the obtained results underline that ANN is a suitable modelling approach for the evaluation of tensile strength of nano TiO${}_{2}$-coated cotton.

## Figures and Tables

**Table 1:** The input variables for experimental design.

Sample	TiO${}_{2}$ Dosage [g·L${}^{-1}$]	Temperature [°C]	UV Irradiation Time [min]
1	6	70	80
2	4	30	120
3	6	45	80
4	6	45	15
5	8	30	40
6	4	60	40
7	4	30	40
8	8	60	40
9	6	20	80
10	2	45	80
11	8	60	120
12	8	30	120
13	10	45	80
14	6	45	150
15	4	60	120

**Table 2:** Parameters of training network.

Parameters	Settings
Transfer function of hidden layers	tansig, tansig
Transfer function of output layer	tansig
Training function	trainbr
Performance goal	0.00001
Input node	3
Output node	2
Number of hidden nodes	12, 12
Epochs	1000

**Table 3:** Errors of ANN, MLR and PRA.

Functional Properties	Methods	MAE	MSE	RMSE	MAPE	R${}^{2}$
Nano TiO${}_{2}$-coated amount after UV treatment	ANN (training)	2.94	20.18	4.52	0.23	1
	ANN (testing)	2.86	20.05	4.47	0.22	0.99
	MLR	147.66	$2.95\times 10^{5}$	542.85	7.24	0.67
	PRA	202.22	$8.26\times 10^{4}$	287.41	13.18	0.87
Tensile strength after UV treatment	ANN (training)	1.121	3.011	1.730	0.407	0.99
	ANN (testing)	1.112	2.978	1.7257	0.2348	1
	MLR	6	83.6	9.1433	1.2703	0.90
	PRA	6.90	67.73	8.23	1.48	0.92

**Table 4:** Analysis report of experimental and predicted values of nano TiO${}_{2}$ coated amount and tensile strength under ANN, MLR and PRA models.

Functional Properties	Methods	*p*-Value	F-Value
Nano TiO${}_{2}$-coated amount on cotton after UV treatment	ANN	0.0012	10.31
	MLR	0.0024	9.92
	PRA	0.0041	9.17
	Experimental	0.0024	9.83
Tensile strength after UV treatment	ANN	0.2163	1.98
	MLR	0.3019	1.39
	PRA	0.34	1.23
	Experimental	0.2525	1.59

## Data Availability

Not applicable.
